# Supplementary material for: Diagnostic and Prognostic Comparison of Immune-Complex-Mediated Membranoproliferative Glomerulonephritis and C3 Glomerulopathy
Source: Cells. 2023 Feb 23;12(5):712. doi: 10.3390/cells12050712 (PMC10000503; doi:10.3390/cells12050712)
Supplement: Supplementary file 1 [file cells-12-00712-s001.zip › cells-2164488-supplementary.pdf]

SUPPLEMENTARY MATERIAL

Supplementary Table S1. Full list of excluded diseases not fulfilling diagnostic criteria for IC-MPGN or C3G or index biopsy only showing MPGN-type of pattern as a secondary damage to another known disease/condition or poor/inadequate biopsy sample.

- Thrombotic microangiopathy in graft's 0-biopsy or due to antibody-mediated rejection
- Systemic lupus nephritis
- Nephrectomy due to cancer
- Vasculitis
- IgA nephropathy
- End-stage renal disease not possible to classify further
- Focal segmental glomerulosclerosis
- Thin basement membrane disease
- Diabetic nephropathy
- Membranous glomerulonephritis
- Transplant rejection
- Fibrillary glomerulonephritis
- Immunotactoid glomerulonephritis
- Cryoglobulinemia
- Self-limiting poststreptococcal glomerulonephritis
- Hypertension induced secondary global and segmental glomerulosclerosis
- Inadequate information and biopsy material for diagnosis
- Unspecified MPGN diagnosis that could not be classified further

Supplementary Table S2. Blood complement analysis performed on patients attending the research outpatient visit.

Complement protein levels:

plasma Factor B
plasma Factor H
serum C3
serum C4
Factor H related protein 1-3 (FHR1-3)

Complement functional assays:

serum complement deficiency test (s-C-DEF) including the activities of lectin, classical and alternative pathyways s-CH100CL, s-CH100L and s-CH100Al

Autoantibodies:

serum C3 nephritic factor (C3nef)
anti-factor H
anti-factor B
anti-C3b

Supplementary Table S3. Immunofluorescence and electron microscopy findings of index biopsies. Values are expressed as means (SD) or number (percentage) of patients. Number of patients signifies the maximum number of patients with the information.

	IC-MPGN n = 37	C3G n = 23	p-value
**Immunofluorescence staining**			
Intensity of IgG staining			
0, n (%)	9 (24)	19 (83)	<0.001
1, n (%)	0 (0)	0 (0)	1.000
2, n (%)	4 (11)	4 (17)	0.468
3, n (%)	13 (35)	0 (0)	<0.001
4, n (%)	11 (30)	0 (0)	0.004
Intensity of IgA staining			
0, n (%)	25 (75), n = 36	21 (91)	0.059
1, n (%)	4 (11), n = 36	0 (0)	0.149
2, n (%)	6 (17), n = 36	2 (9)	0.464
3, n (%)	1 (3), n = 36	0 (0)	1.000
4, n (%)	0 (0), n = 36	0 (0)	1.000
Intensity of IgM staining			
0, n (%)	16 (43)	18 (78)	0.015
1, n (%)	4 (11)	3 (13)	1.000
2, n (%)	10 (27)	2 (9)	0.107
3, n (%)	5 (14)	0 (0)	0.146
4, n (%)	2 (5)	0 (0)	0.519
Intensity of C3 staining			
0, n (%)	1 (3)	0 (0)	1.000
1, n (%)	1 (3)	0 (0)	1.000
2, n (%)	10 (27)	0 (0)	0.009
3, n (%)	14 (38)	11 (48)	0.519
4, n (%)	11 (30)	12 (52)	0.105
Intensity of C1q staining			
0, n (%)	6 (17), n = 35	17 (74)	<0.001
1, n (%)	2 (6), n = 35	1 (4)	1.000
2, n (%)	10 (29), n = 35	5 (22)	0.760
3, n (%)	10 (29), n = 35	0 (0)	0.004
4, n (%)	7 (20), n = 35	0 (0)	0.035
**Electron microscopy changes**			
Amount of subepithelial deposits			
None, n (%)	23 (79), n=29	10 (53), n=19	0.482
Few, n (%)	4 (14), n=29	6 (32), n=19	0.302
Moderate, n (%)	1 (4), n=29	0 (0), n=19	1.000
Many, n (%)	1 (4), n=29	3 (16), n=19	0.299
Amount of subendothelial deposits			
None, n (%)	3 (10), n=29	8 (42), n=19	0.091
Few, n (%)	11 (38), n=29	5 (26), n=19	0.766
Moderate, n (%)	8 (28), n=29	3 (16), n=19	0.512
Many, n (%)	7 (24), n=29	3 (16), n=19	0.727
Amount of intramembranous deposits			
None, n (%)	26 (90), n=29	14 (74), n=19	0.825
Few, n (%)	3 (10), n=29	3 (16), n=19	0.678
Moderate, n (%)	0 (0), n=29	1 (5), n=19	0.408
Many, n (%)	0 (0), n=29	1 (5), n=19	0.408
Amount of mesangial deposits			
None, n (%)	8 (28), n=29	9 (47), n=19	0.4
Few, n (%)	12 (41), n=29	7 (37), n=19	1.000
Moderate, n (%)	6 (21), n=29	1 (5), n=19	0.402
Strong, n (%)	3 (10), n=29	2 (11), n=19	1.000

Supplementary Table S4. Multivariate analysis for histological determinants of progressive disease.

Variable	OR	95% CI	p-value
**Mesangial hypercellularity** [1]			
IC-MPGN	0.6	0.1-3.0	0.538
C3G	0.9	0.3-3.5	0.936
**Endocapillary hypercellularity** [1]			
IC-MPGN	1.2	0.2-10.2	0.841
C3G	0.5	0.03-6.4	0.560
**Crescents** [1]			
IC-MPGN	1.4	0.8-2.3	0.221
C3G	0.7	0.3-1.7	0.463
**C3 staining** [1]			
IC-MPGN	0.6	0.3-1.2	0.165
C3G	2.6	0.6-11.6	0.218
**IgG staining** [1]			
IC-MPGN	0.7	0.2-1.9	0.459
C3G	0.3	0.04-2.9	0.318
**Tubular atrophy** [2]			
IC-MPGN	0.4	0.03-6.0	0.505
C3G	4.2e+26	0 - inf	0.999
**Interstitial fibrosis** [2]			
IC-MPGN	0.7	0.05-10.7	0.810
C3G	3.9e+9	0 - inf	1.000
**Sclerotic glomeruli** [2]			
IC-MPGN	1.1	0.8-1.4	0.572
C3G	1.3	0 - inf	1.000
**Double contours** [2]			
IC-MPGN	1.4e+7	0 - inf	0.996
C3G	2.1e+8	0 - inf	1.000

[1] Grouped together in a multivariate analysis,
[2] Grouped together in a multivariate analysis
inf = infinite

Supplementary Table S5. Multivariate analysis for baseline clinical and laboratory determinants of progressive diseases.

Variable	OR	95% CI	p-value
**Daily urine protein excretion** [1]			
IC-MPGN	0.9	0.6-1.2	0.419
C3G	2.0	0.9-4.4	0.080
**eGFR** [1]			
IC-MPGN	1.0	0.9-1.0	0.106
C3G	0.9	0.8-1.0	0.128
**Serum albumin** [1]			
IC-MPGN	1.2	0.9-1.6	0.325
C3G	1.7	1.1-2.8	0.03
**Hematuria** [1]			
IC-MPGN	0.3	0.01-8.3	0.515
C3G	NA*	NA*	NA*
**Hypertension** [1]			
IC-MPGN	0.93	0.38-2.29	0.648
C3G	0.98	0.17-1.18	0.270
**Age** [2]			
IC-MPGN	1.0	0.9-1.1	0.992
C3G	0.9	0.9-1.0	0.084
**Gender** [2]			
IC-MPGN	3.3	0.3-34.6	0.318
C3G	1.5	0.2-11.2	0.666
**Body mass index** [2]			
IC-MPGN	0.9	0.7-1.2	0.550
C3G	1.1	0.9-1.5	0.310

[1] Grouped together in a multivariate analysis,

[2] Grouped together in a multivariate analysis

eGFR = estimated glomerular filtration rate, calculated according to the Chronic Kidney Disease Epidemiology Collaboration (CKD-EPI) equation, NA = not applicable

*All cases have hematuria in C3G group
